# Association of Alzheimer’s and Lewy body disease pathology with basal forebrain volume and cognitive impairment

**DOI:** 10.1186/s13195-025-01678-x

**Published:** 2025-01-27

**Authors:** Julia Schumacher, Stefan Teipel, Alexander Storch

**Affiliations:** 1 Department of Neurology, University Medical Center Rostock, 18147 Rostock, Germany; 2 Deutsches Zentrum für Neurodegenerative Erkrankungen (DZNE) Rostock-Greifswald, 18147 Rostock, Germany; 3 Department of Psychosomatic Medicine, University Medical Center Rostock, 18147 Rostock, Germany

## Abstract

**Background:**

Degeneration of the basal forebrain cholinergic system is a hallmark feature shared by Alzheimer’s disease (AD) and Lewy body disease (LBD) whereas hippocampus atrophy is more specifically related to AD. We aimed to investigate the relationship between basal forebrain and hippocampus atrophy, cognitive decline, and neuropathology in a large autopsy sample.

**Methods:**

Data were obtained from the National Alzheimer’s Coordinating Center (NACC). Basal forebrain and hippocampus volumes were extracted using an established automated MRI volumetry approach. Associations of regional volumes with pathological markers (Braak stage, CERAD score, and McKeith criteria for LB pathology) and cognitive performance were assessed using Bayesian statistical methods.

**Results:**

We included people with autopsy-confirmed pure AD (*N* = 248), pure LBD (*N* = 22), and mixed AD/LBD (*N* = 185). Posterior basal forebrain atrophy was most severe in mixed AD/LB pathology compared to pure AD (Bayes factor against the null hypothesis BF_10_ = 16.2) or pure LBD (BF_10_ = 4.5). In contrast, hippocampal atrophy was primarily associated with AD pathology, independent of LB pathology (pure AD vs. pure LBD: BF_10_ = 166, pure AD vs. mixed AD/LBD: BF_10_ = 0.11, pure LBD vs. mixed AD/LBD: BF_10_ = 350). Cognitive performance was more impaired in AD pathology groups, with Braak stage being the strongest predictor. Hippocampal volume partially mediated this relationship between tau pathology and cognitive impairment, while basal forebrain volume had a limited role in mediating the relationship between pathological burden and cognitive outcomes.

**Conclusion:**

In a heterogeneous autopsy sample, AD and LB pathology both contribute to cholinergic basal forebrain degeneration whereas hippocampus atrophy is more specifically related to AD pathology. Cognitive deficits are primarily associated with tau pathology which is partly mediated by hippocampus, but not basal forebrain atrophy.

**Supplementary Information:**

The online version contains supplementary material available at 10.1186/s13195-025-01678-x.

## Introduction

Degeneration of the basal forebrain cholinergic system is a hallmark feature shared by Alzheimer’s disease (AD) and Lewy body disease (LBD) [1, 2]. Most previous studies in this area have investigated basal forebrain volume in clinically defined cases with AD dementia or dementia with Lewy bodies (DLB). They have consistently found the basal forebrain to degenerate early in both conditions, particularly its posterior part which encompasses the nucleus basalis of Meynert (NBM) [3, 4]. These cholinergic changes are related to cognitive impairment and are predictive of future cognitive decline [3, 5]. Some studies have suggested that cholinergic loss might be even more pronounced in LBD compared to AD [6], and the effectiveness of cholinergic remediation with cholinesterase inhibitors might be higher in LBD [7]. 

Despite these consistent neuroimaging and clinical findings, the neuropathological correlates of basal forebrain atrophy in AD and LBD are less widely studied and less well understood. Neuropathologically, LBD is defined by the presence of intracellular alpha-synuclein aggregates in the form of Lewy bodies and neurites while AD is defined by extracellular amyloid plaques and intracellular tau neurofibrillary tangles. However, both diseases are characterized by large pathological heterogeneity and there is significant overlap between them. AD co-pathology is common in LBD [8, 9] and this additional pathological burden has been associated with higher atrophy rates [10, 11], lower cognitive performance [12], and more rapid cognitive decline [13, 14]. Conversely, LB co-pathology also occurs in AD and is associated with more severe motor and frontal-dysexecutive impairment [15, 16]. In a considerable number of individuals the amount of co-pathology is so severe that they meet pathological diagnostic criteria for both AD and LBD at autopsy [17]. 

One previous study has investigated the association between AD and LB pathology and basal forebrain atrophy in the Alzheimer’s Disease Neuroimaging Initiative (ADNI) dataset and found basal forebrain degeneration to be associated with the presence of global LB pathology and cortical but not local amyloid plaque load [18]. However, this study used a relatively small sample (*N* = 62) which only comprised people with a clinical diagnosis of mild cognitive impairment (MCI) or AD dementia in addition to cognitively unimpaired controls. In the present study, we expand on this topic by including a larger and more heterogeneous autopsy sample of people with varying degrees of AD and LB pathology, including those with mixed disease. We thereby sought to investigate the three-way association between the different pathologies, basal forebrain volume, and cognition in individuals along the AD-LBD pathological spectrum. We compared findings for the basal forebrain with the more widely studied hippocampus as a region that also degenerates early in the course of AD, but which is generally relatively spared in LBD [10]. 

## Methods

### Participants

Data used in this analysis were obtained from the National Alzheimer’s Coordinating Center (NACC) database (https://naccdata.org). The NACC Uniform Data Set (UDS) was created in 2005 to collect standard clinical data on participants with any level of cognition at NIA-funded Alzheimer’s Disease Research Centers (ADRCs), approximately on an annual basis [19, 20]. These data include detailed participant demographics, family and health history, physical and neurological exam findings, behavioural and functional assessments, and a multi-domain neuropsychological test battery. Additionally, the NACC Neuropathology dataset contains autopsy data for a subset of participants who have died and consented to autopsy and a subset of participants also underwent MR imaging. This analysis used data from 19 ADRCs for UDS visits conducted between September 2005 and November 2022. We included all participants that had at least one clinical visit, good quality MRI data, and a post-mortem assessment of AD and LB pathology (details below).

The inclusion of de-identified data in the NACC database was approved by the Institutional Review Board at each participating ADRC and all participants provided informed consent at the time of enrolment at the individual ADRCs.

### Demographics and clinical characteristics

We report demographics and clinical characteristics based on the last UDS visit prior to autopsy. Participants were assessed for the presence of cognitive impairment at each visit and assigned to one of the following categories: normal cognition, cognitively impaired without meeting criteria for MCI, MCI or dementia. The diagnostic method depends on the routine practice at each ADRC, but followed standard diagnostic guidelines. For all participants without normal cognition, a presumptive etiological diagnosis of the cognitive disorder was made by the clinician(s).

### Neuropathological assessment

Autopsy evaluations for the NACC cohort were conducted at each of the participating ADRCs, following consensus guidelines, but according to each center’s own protocol which can differ between sites [21]. AD pathology was defined by the Consortium to Establish a Registry for Alzheimer’s Disease (CERAD) score of neuritic plaque density (none, sparse, moderate or frequent) [22] and Braak stage for tau neurofibrillary tangles (ranging from 0-VI) [23]. LB pathology was assessed according to the McKeith et al. criteria categorizing LB pathology into brainstem-predominant, limbic or amygdala-predominant, and neocortical [24]. Participants where Lewy bodies were found to be present, but the region was unspecified, were excluded from the present analysis. Based on CERAD score, Braak stage, and LB criteria, participants were divided into three pathologically defined groups irrespective of their clinical diagnosis. Individuals were classified as having AD if they showed at least intermediate AD neuropathologic change according to the National Institute on Aging–Alzheimer’s Association guidelines for the neuropathologic assessment of AD [25].

1. Pure AD: moderate/frequent neuritic plaques (CERAD score C2 or C3) & Braak stage III-VI & no LB pathology in any region,
2. Pure LBD: no/sparse neuritic plaques (CERAD score C0 or C1) & Braak stage 0-II & LB pathology present in any region,
3. Mixed AD/LBD: moderate/frequent neuritic plaques (CERAD score C2 or C3) & Braak stage III-VI & LB pathology present in any region.

Since information on Thal phase for amyloid plaques has only been included in the NACC protocol from 2014 onwards and was missing for about 1/4 of cases included in this study, this was not used for the pathological classification.

### Neuropsychological testing

Participants underwent a multi-domain neuropsychological assessment including measures of attention, processing speed, executive function, episodic memory, and language [26]. Different test batteries were used in UDS version 2 (from September 2005 to March 2015) and UDS version 3 (from 2015 onwards). We used the conversion tables estimated in a crosswalk study [27] to convert the new scores from UDS 3 visits to equivalent scores on the previously used tests from UDS 2 to combine them for analysis.

The Clinical Dementia Rating (CDR^®^ Dementia Staging Instrument) and Mini Mental State Examination (MMSE) were used for overall cognition. Episodic memory was tested with the Logical Memory Test from the Wechsler Memory Scale – Revised (WMS-R) including immediate story recall and delayed recall. Attention was assessed using the WMS-R’s digit span forward and backward tests. The Boston Naming Test was included as a measure of language function. Verbal fluency was assessed by the total number of animals/vegetables named in 60 s. The difference between the total time to complete Trail Making Test B and A was used as a measure of executive function/attention. Finally, processing speed was tested with the Digit Symbol Test from the Wechsler Adult Intelligence Scale – Revised. Raw scores were used for all cognitive scores. However, covariates for age, sex, and years of education were included in all analyses to account for the influence of these variables.

### MRI data acquisition and processing

**Fig. 1:**
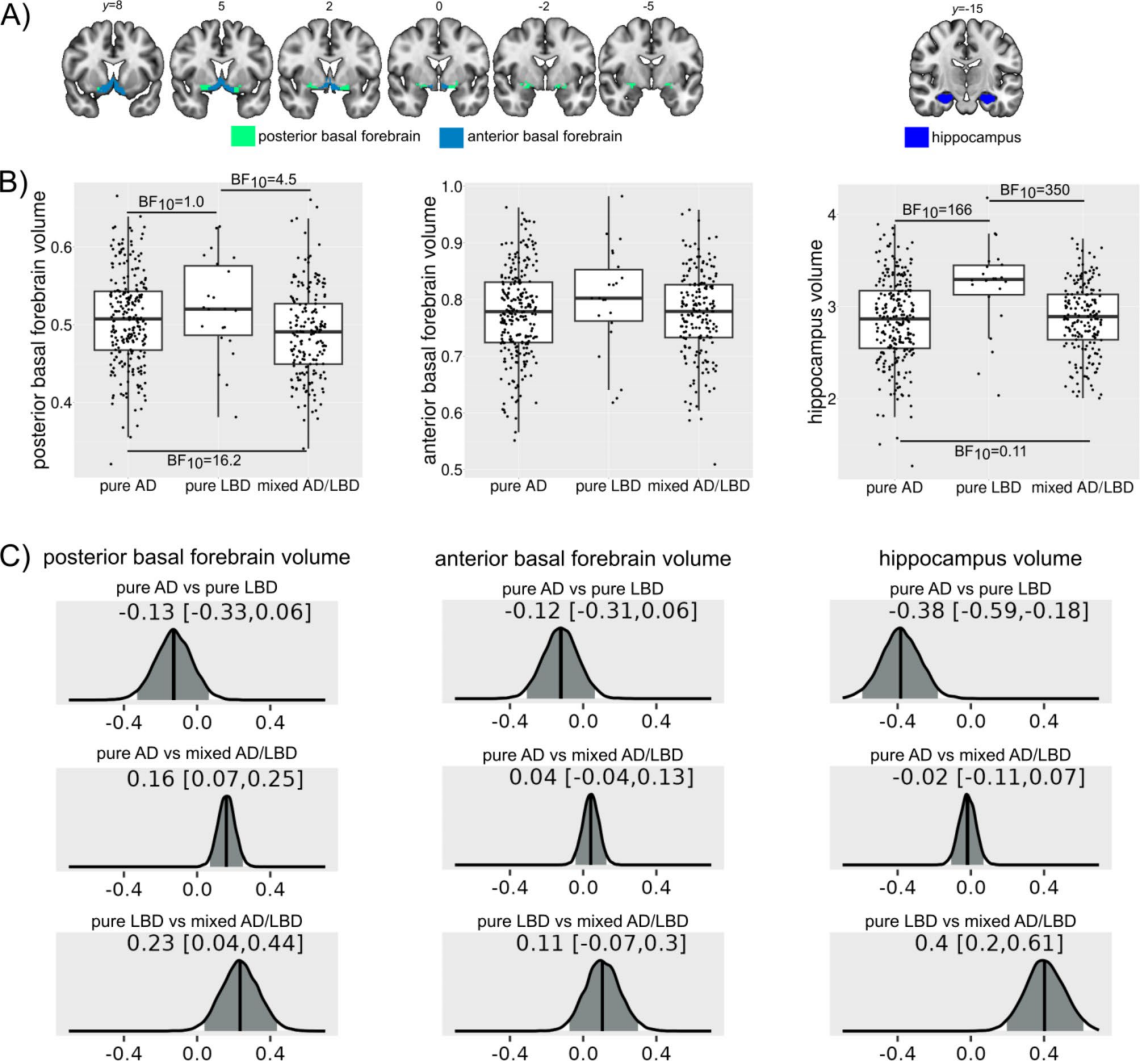
Group comparison of regional brain volumes. (**A**) Masks that were used for the extraction of volumes from the anterior and posterior basal forebrain and the hippocampus. (**B**) Bayes factors quantifying evidence against the null hypothesis (BF_10_) from Bayesian ANCOVAs including covariates for age at autopsy, sex, years of education, time interval between MRI and autopsy, and site. In each box plot the central line corresponds to the sample median, the upper and lower border of the box represent the 25th and 75th percentile, respectively, and the length of the whiskers corresponds to 1.5x the interquartile range. All regional volumes are normalized with respect to total intracranial volume. (**C**) Posterior distributions of the parameter estimates for the effect of group in the Bayesian ANCOVA models. The median is marked with a solid line and the 95% credible intervals in grey, and stated in each plot

T1-weighted 3D volumetric MR images in the NACC database were acquired on different 1.5 or 3T scanners. The CAT12 toolbox in SPM (http://www.fil.ion.ucl.ac.uk/spm/) was used to segment T1-weighted MR images into grey matter, white matter, and cerebrospinal fluid, and spatially normalize them to MNI space. Voxel values of spatially normalized grey matter maps were modulated by the Jacobian determinant of the deformation parameters to preserve the volume present in native space. Basal forebrain volumes were estimated by summing the modulated grey matter values within a consensus ROI combining information from existing cytoarchitectonic maps of basal forebrain cholinergic nuclei in MNI space, which have been derived from combined histology and MRI of post-mortem brains [28–31]. We estimated the volume of two functionally defined basal forebrain sub-regions that were identified based on their differential cortical connectivity profile in resting-state fMRI data [31]. In this subdivision, the posterior basal forebrain mainly corresponds to the cytoarchitectonic sub-region of the NBM (anterior-lateral, intermediate and posterior parts) while the anterior basal forebrain covers the medial septum and diagonal band of Broca as well as the anterior-medial parts of the NBM (see Fig. 1A).

Hippocampal volumes were estimated from T1-weighted MR images using an analogous automated volumetry approach based on a consensus MNI template of the hippocampus according to the European Alzheimer’s Disease Consortium and Alzheimer’s Disease Neuroimaging Initiative (EADC-ADNI) Harmonized Protocol [32]. Regional volumes were normalized with respect to total intracranial volume and averaged across left and right hemispheres.

### Statistics

Statistical analyses were performed in a Bayesian framework using Jeffrey’s Amazing Statistics Program (JASP, version 0.17.1) and the BayesFactor package (version 0.9.12) in R (https://www.r-project.org/). We report the Bayes Factor (BF_10_) to quantify evidence in favour of the alternative over the null hypothesis [33] and the posterior distributions of parameter estimates. Numerical accuracy was established with 10,000 iterations using a Markov Chain Monte Carlo algorithm. The Bayes Factor is interpreted as the relative likelihood of the data under the models at hand, i.e. BF_10_ quantifies the likelihood of the data given H1 compared to the likelihood of the data given H0. According to Bayesian analysis reporting guidelines in JASP, a BF_10_ between 3 and 10 indicates a moderate, a BF_10_ between 10 and 30 indicates a strong, a BF_10_ between 30 and 100 indicates a very strong, and a BF_10_ > 100 indicates an extreme level of evidence in favour of the alternative model over the null model. Equivalently, if BF_10_ is below 1/3, 1/10, 1/30, 1/100, it indicates a moderate, strong, very strong, or extreme level of evidence, respectively, in favour of the null over the alternative hypothesis. Thus, a key strength of Bayesian hypothesis testing as opposed to the frequentist approach is that it provides the possibility to directly quantify support in favour of the null hypothesis, not only against it [33]. 

Basal forebrain and hippocampal volumes were compared between the three pathologically defined groups using Bayesian ANCOVAs. To further test associations between regional brain volume and the severity of the different pathologies, we used Bayesian ANCOVAs predicting regional volumes from the pathological scales (Braak stage, CERAD score, presence of LB pathology). Both analyses included covariates for age at autopsy, sex, years of education, time interval between MRI and autopsy, and site.

To test whether regional brain volumes differed systematically between different scanners, we performed Bayesian ANCOVAs for the effect of site on basal forebrain and hippocampus volumes including covariates for age, sex, years of education, time interval between MRI and autopsy, and pathological group.

Differences in cognitive scores between the pathological groups were assessed using Bayesian ANCOVAs. Associations between the severity of pathology and cognition were assessed with Bayesian ANCOVAs including the respective cognitive score as dependent variable and the three pathological scales as predictors. These analyses included covariates for age, sex, years of education, and time between clinical visit and autopsy.

To test how much of the association between pathology and cognitive scores was mediated by regional grey matter volume, we conducted one Bayesian mediation analysis per cognitive test with three predictors (Braak stage, CERAD score, presence of LB pathology), two mediators (posterior basal forebrain volume and hippocampus volume) and covariates for age, sex, years of education, time interval between MRI and autopsy, and site, using the R package blavaan (version 0.5.4).

## Results

### Demographics

A total of 638 participants from the NACC database fulfilled inclusion criteria and were considered for analysis. 91 participants were excluded because they did not fall into any of the three pathological groups (i.e. high CERAD score with low Braak stage or vice versa) and a further 92 participants had no evidence of AD or LB pathology. Thus, the final cohort included 248 people with pure AD, 22 with pure LBD, and 185 with mixed AD/LB pathology (see Table 1). Age at death, time between the last clinical visit/the last MRI and autopsy as well as years of education, percentage of carriers of the APOE ε4 allele, and overall levels of cognitive impairment and depression were similar in all groups. Clinically, most participants in the pure AD and mixed AD/LBD groups were diagnosed with AD whereas only 62% of the pure LBD group had received a clinical LBD diagnosis.

The majority of people in the pure AD group had Braak stage V or VI and about 65% had frequent neuritic plaques. In the pure LBD group, the distribution of participants among the brainstem-predominant, limbic, and neocortical groups was approximately 1/3 each. In the mixed AD/LBD group most people showed limbic or neocortical LB pathology, Braak stage V or VI and frequent neuritic plaques. The presence of co-occurring vascular pathology was very common across all groups and the severity of TDP-43 co-pathology did not differ between the groups.

**Table 1:** Demographic and clinical information

		Pure AD (*N* = 248)	Pure LBD (*N* = 22)	Mixed AD/LBD (*N* = 185)	Group differences
Age at death		82.0 (10.1)	80.1 (11.4)	79.7 (9.3)	BF_10_ = 0.62^a^
Last visit - death (years)		1.8 (1.8)	1.4 (1.3)	1.9 (2.2)	BF_10_ = 0.09^a^
Last MRI - death (years)		5.1 (3.1)	5.1 (3.0)	5.2 (3.0)	BF_10_ = 0.04^a^
Male: female, N (%)		131:117 (53%:47%)	19:3 (86%:14%)	117:68 (63%:37%)	BF_10_ = 5.8^b^
Years of education		15.5 (3.0)	15.7 (3.7)	15.4 (3.2)	BF_10_ = 0.05^a^
APOE ε4 allele, N (%)		143 (61%)	6 (30%)	108 (60%)	BF_10_ = 0.43^c^
Cognitive status, N (%)					BF_10_ = 0.25^d^
	normal cognition	7 (2.8%)	1 (5%)	2 (1%)	
	impaired-not-MCI	2 (0.8%)	0	0	
	MCI	22 (8.9%)	2 (9%)	6 (3%)	
	dementia	217 (88%)	19 (86%)	177 (96%)	
Primary etiological diagnosis based on clinical information^e^					
	AD	209 (87%)	7 (33%)	151 (83%)	
	LBD	12 (5%)	13 (62%)	22 (12%)	
	Vascular impairment	8 (3%)	0	3 (2%)	
	FTLD	4 (2%)	0	3 (2%)	
	other	6 (3%)	1 (5%)	2 (1%)	
CDR global		1.84 (0.96)	1.59 (0.93)	1.95 (0.92)	BF_10_ = 0.19^a^
Geriatric depression scale		2.2 (2.3)	3.2 (2.0)	2.6 (2.7)	BF_10_ = 0.26^a^
Neuropath findings, N (%)					
	Braak stage III/IV	41 (17%)	-	36 (19%)	
	Braak stage V/VI	207 (83%)	-	149 (81%)	
	Moderate NP	86 (35%)	-	58 (31%)	
	Frequent NP	162 (65%)	-	127 (69%)	
	Brainstem-predominant LB	-	6 (27%)	19 (10%)	
	Limbic or amygdala-predominant LB	-	8 (36%)	93 (50%)	
	Neocortical LB	-	8 (36%)	73 (40%)	
	Presence of vascular pathology	244 (98%)	22 (100%)	183 (99%)	BF_10_ = 0.26^f^
	TDP-43 sum score	0.84 (1.36)	0.89 (1.27)	0.95 (1.26)	BF_10_ = 0.10^g^

Mean (standard deviation), if not otherwise specified. All clinical information from last visit before death

^a^ Bayesian ANOVA

^b^ Bayesian contingency tables test (independent multinomial); post-hoc pairwise comparisons: pure AD vs. pure LBD: BF_10_ = 10.9, pure AD vs. mixed AD/LBD: BF_10_ = 1.3, pure LBD vs. mixed AD/LBD: BF_10_ = 1.2

^c^ Bayesian contingency tables test (independent multinomial); available for 235 pure AD, 20 pure LBD, and 179 mixed AD/LBD

^d^ Bayesian contingency tables test (independent multinomial)

^e^ only applicable to those without normal cognition; information missing for 2 pure AD, and 2 mixed AD/LBD

^f^ presence of ischemic, hemorrhagic or vascular pathology; Bayesian contingency tables test (independent multinomial); available for 247 pure AD, 22 pure LBD, 184 mixed AD/LBD

^g^ score from 0–4 calculated as the sum of regional TDP-43 scores in amygdala, hippocampus, entorhinal/inferior temporal cortex and neocortex; Bayesian ANOVA; available for 96 pure AD, 9 pure LBD, 77 mixed AD/LBD

CDR, Clinical Dementia Rating; LB, Lewy bodies; MCI, mild cognitive impairment; NP, neuritic plaques

**Table 2:** Group comparison of regional brain volumes

	Pure AD	Pure LBD	Mixed AD/ LBD	Overall group comparison	Pairwise comparisons		
					Pure AD vs. pure LBD	Pure AD vs. mixed	Pure LBD vs. mixed
Posterior BF volume	0.507 (0.059)	0.522 (0.064)	0.489 (0.057)	BF_10_ = 32.8	BF_10_ = 1.0	BF_10_ = 16.2	BF_10_ = 4.5
Anterior BF volume	0.777 (0.080)	0.799 (0.094)	0.774 (0.075)	BF_10_ = 0.16	BF_10_ = 0.9	BF_10_ = 0.14	BF_10_ = 0.58
Hippocampus volume	2.85 (0.46)	3.21 (0.49)	2.86 (0.38)	BF_10_ = 41.5	BF_10_ = 166.0	BF_10_ = 0.11	BF_10_ = 350

Mean (standard deviation) and group comparison by Bayesian ANCOVAs including covariates for age at MRI, sex, years of education, time between MRI and autopsy, and site. Volumes are presented as the ratio between regional grey matter volume and total intracranial volume

AD, Alzheimer’s disease; BF, basal forebrain; BF_10_, Bayes factor in favor of H1 over H0; LBD, Lewy body disease

### Volumetric analyses

We found moderate to strong evidence for smaller posterior basal forebrain volumes in the mixed AD/LBD compared to the pure AD (BF_10_ = 16.2) and pure LBD (BF_10_ = 4.5) groups whereas the evidence for the comparison between the pure AD and pure LBD groups was inconclusive (BF_10_ = 1.0, Table 2; Fig. 1). For anterior basal forebrain volume there was evidence in favour of the null hypothesis, i.e. that the three groups did not differ (BF_10_ = 0.16). Hippocampus volumes were larger in the pure LBD group compared to the pure AD (BF_10_ = 166.0) and the mixed AD/LBD groups (BF_10_ = 350), while evidence was against a difference between AD and mixed AD/LBD cases (BF_10_ = 0.11). Results remained consistent when analysing left and right hemispheres separately (Supplementary Table S1).

There was evidence that site did not have an effect on posterior (BF_10_ = 0.295) and anterior basal forebrain (BF_10_ = 0.284) as well as hippocampus volumes (BF_10_ = 0.184).

When predicting posterior basal forebrain volume the model with the strongest support for H1 contained both LB pathology and Braak stage and was 7.3 times more likely than the model that contained all three pathologies and 33.9 times more likely than the model that contained LB pathology and CERAD scores (Table 3).

There was no evidence for an association between anterior basal forebrain volume and the severity of the three pathologies (Table 3).

For hippocampus volume, the most supported models all included Braak stage with the model that only included Braak stage being 3.6 times more likely than the model that also contained CERAD score and 7.9 times more likely than the model that also included LB pathology (Table 3). The model that only contained LB pathology showed evidence for no association.

**Table 3:** Effect of tau, amyloid, and Lewy body pathology on regional brain volumes

Model	*P*(M)	*P*(M|data)	BF_M_	BF_10_	Error %	Evidence for H1/H0
Posterior basal forebrain volume						
Null model	0.25	0.003	0.01	1.0		
Lewy + Braak	0.083	0.68	36.2	664.6	3.7	Extreme for H1
Lewy + Braak + CERAD	0.25	0.28	0.90	90.5	3.4	Very strong for H1
Lewy + CERAD	0.083	0.02	0.18	19.6	3.9	Strong for H1
Lewy	0.083	0.01	0.10	10.7	6.9	Strong for H1
Braak	0.083	0.006	0.05	5.8	2.3	Moderate for H1
Braak + CERAD	0.083	5.6*10^− 4^	0.005	0.55	3.1	Weak for H0
CERAD	0.083	4.6*10^− 4^	0.004	0.45	3.1	Weak for H0
Anterior basal forebrain volume						
Null model	0.25	0.64	4.3	1.0		
CERAD	0.083	0.22	4.5	1.03	3.7	Weak for H1
Braak	0.083	0.04	0.57	0.20	1.4	Moderate for H0
CERAD + Lewy	0.083	0.04	0.49	0.17	5.6	Moderate for H0
Lewy	0.083	0.03	0.37	0.13	2.2	Moderate for H0
Braak + CERAD	0.083	0.01	0.14	0.05	1.8	Very strong for H0
Braak + Lewy	0.083	0.009	0.11	0.04	7.5	Very strong for H0
Braak + CERAD + Lewy	0.25	0.007	0.03	0.01	2.5	Very strong for H0
Hippocampus volume						
Null model	0.25	4.8*10^− 6^	1.2*10^− 5^	1.0		
Braak	0.083	0.67	16.3	422,422		Extreme for H1
Braak + CERAD	0.083	0.19	1.7	116,617		Extreme for H1
Braak + Lewy	0.083	0.09	0.68	53,193		Extreme for H1
Braak + CERAD + Lewy	0.25	0.06	0.14	11,497		Extreme for H1
CERAD	0.083	9.0*10^− 4^	0.007	565.2		Extreme for H1
CERAD + Lewy	0.083	9.6*10^− 5^	7.0*10^− 4^	60.2		Very strong for H1
Lewy	0.083	4.8*10^− 7^	3.5*10^− 6^	0.30		Moderate for H0

Results from Bayesian ANCOVAs across all groups with the respective regional volume measure as dependent variable and the three neuropathological staging systems (Braak stage, CERAD score, presence of Lewy body pathology) as predictors including covariates for age at MRI, sex, years of education, time between MRI and autopsy, and site. Covariates are included in all models (including the null model)

BF_10_, Bayes factor in favour of H1 over H0; BF_M_, degree to which the data have changed the prior model odds; error %, numerical stability of BF_10_ over 10 000 Markov Chain Monte Carlo iterations; H0, null hypothesis; H1, alternative hypothesis; P(M), model’s prior probability; P(M|data), model’s posterior probability after observing the data

### Associations with cognitive scores

There was evidence for group differences for the MMSE, Boston Naming Test, immediate and delayed recall, and the two verbal fluency tests with the pure LBD group performing better than the pure AD and mixed AD/LBD groups and no difference between the latter two (Table 4, Supplementary Figure S1). The association between pathological scores and cognitive performance revealed that Braak staging was the strongest predictor for all cognitive tests except the Trail Making Test. In contrast, the models that only included LB pathology as predictor generally showed evidence for no association with cognition (Table 5).

**Fig. 2:**
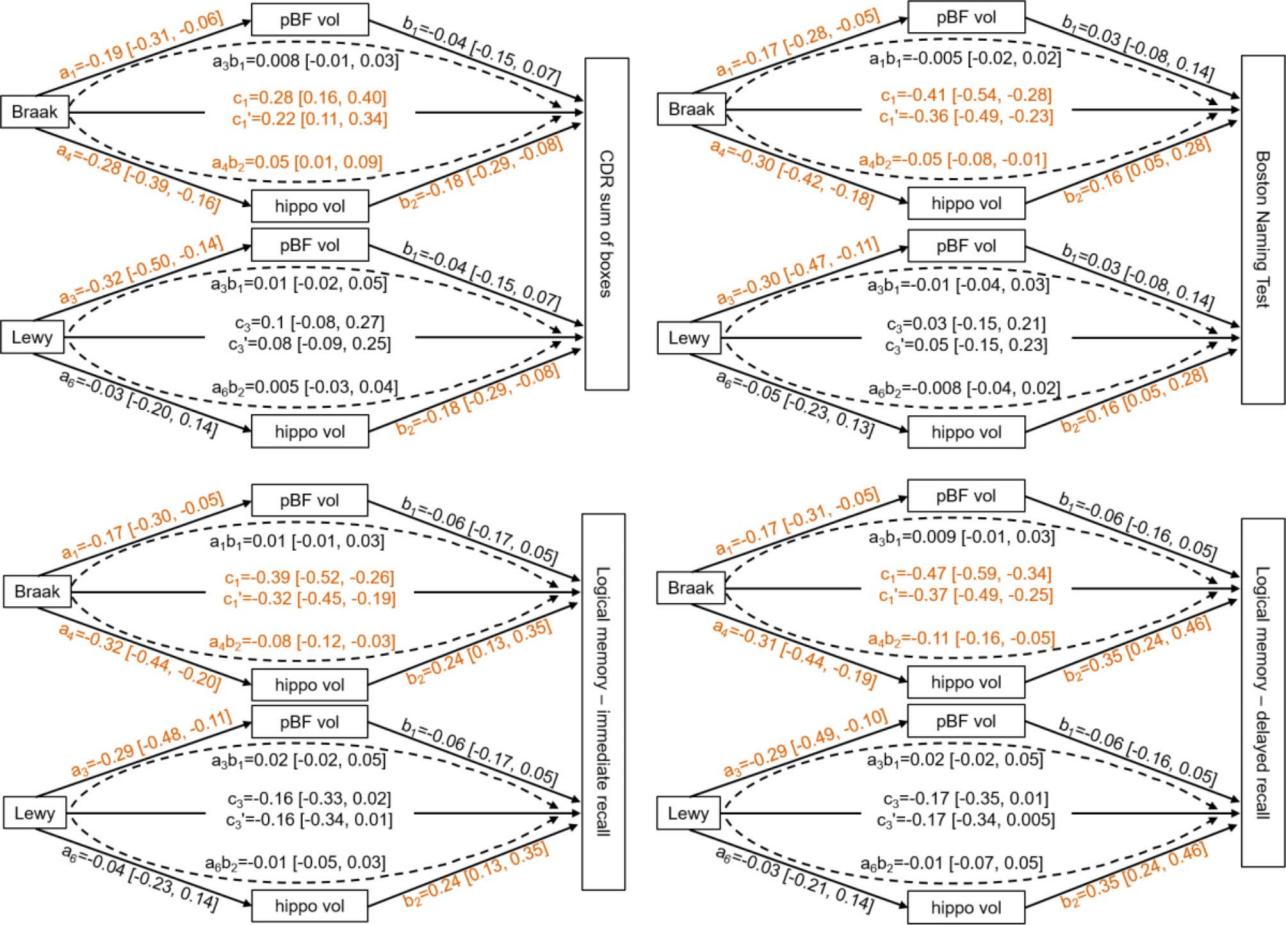
Results from mediation analyses. One mediation analysis was conducted per cognitive test with three predictors (Braak stage, CERAD score, presence of Lewy body pathology), two mediators (posterior basal forebrain volume, hippocampus volume) and covariates for age, sex, years of education, time interval between MRI and autopsy, and site. 95% credible intervals of the parameter estimates were estimated. Credible intervals that don’t overlap with zero are marked in orange. Note that CERAD was included in the models, but is not displayed in the figure as none of the effects for CERAD was significant (see Supplementary Figures S2-4 for complete mediation results including all three mediators). c = total effect of pathology on cognition, c’= direct effect of pathology on cognition controlling for volume, a = effect of pathology on volume, b = effect of volume on cognition, a*b = indirect effect of pathology on cognition mediated via volume (= c-c’). CDR, clinical dementia rating; hippo vol, normalised hippocampus volume; Lewy, presence of Lewy body pathology; pBF vol, normalised posterior basal forebrain volume

Results from the mediation analyses can be found in Fig. 2 for those models with a significant mediation (full results in Supplementary Figures S2-4). There was evidence for a direct effect of Braak stage (c_1_) on all cognitive tests except for the Trail Making Test. For CDR sum of boxes, MMSE, Boston Naming Test, and memory scores, part of this association was mediated via hippocampus volume (a_4_b_2_) whereas there was no mediation via posterior basal forebrain volume (a_1_b_1_) for any of the tests.

LB pathology had a total effect on performance on the Trail Making Test; however, this was not mediated via hippocampus or basal forebrain volume. Furthermore, LB pathology had an effect on posterior basal forebrain volume and basal forebrain volume was related to performance on the Digit Symbol Test. There was no evidence for a total effect for CERAD score and no mediation effects for LB pathology or CERAD.

We did not include anterior basal forebrain or the digit span tests in the mediation analyses because they did not show any association with pathological scores in the previous analyses.

**Table 4:** Group comparison of cognition

	Pure AD	Pure LBD	Mixed AD/ LBD	Overall group comparison	Pairwise comparisons		
					Pure AD vs. pure LBD	Pure AD vs. mixed	Pure LBD vs. mixed
CDR SOB	10.9 (5.8)	9.1 (5.3)	11.5 (5.4)	BF_10_ = 0.59	-	-	-
MMSE	16.5 (7.5)	21.2 (4.5)	15.8 (6.3)	BF_10_ = 7.3	BF_10_ = 20.0	BF_10_ = 0.10	BF_10_ = 218.2
Boston Naming	16.1 (8.5)	24.8 (3.1)	16.8 (7.6)	BF_10_ = 421	BF_10_ = 2135	BF_10_ = 0.19	BF_10_ = 1444
Immediate recall	3.2 (3.9)	6.4 (5.6)	2.6 (3.1)	BF_10_ = 368	BF_10_ = 133	BF_10_ = 0.15	BF_10_ = 14,965
Delayed recall	2.1 (3.5)	4.9 (5.3)	1.6 (2.8)	BF_10_ = 88.5	BF_10_ = 65.5	BF_10_ = 0.15	BF_10_ = 2036
Digit span forw. – trials	6.2 (2.6)	7.1 (2.0)	6.2 (2.4)	BF_10_ = 0.25	-	-	-
Digit span forw. – length	5.4 (1.6)	5.8 (1.4)	5.3 (1.4)	BF_10_ = 0.15	-	-	-
Digit span backw. – trials	3.6 (2.1)	4.2 (1.3)	3.4 (2.0)	BF_10_ = 0.11	-	-	-
Digit span backw. – length	3.1 (1.5)	3.5 (0.7)	3.0 (1.4)	BF_10_ = 0.13	-	-	-
Animals	7.4 (5.2)	10.6 (2.9)	6.2 (2.4)	BF_10_ = 4.4	BF_10_ = 16.9	BF_10_ = 0.16	BF_10_ = 206
Vegetables	4.4 (3.8)	5.7 (2.3)	5.3 (1.4)	BF_10_ = 6.1	BF_10_ = 2.7	BF_10_ = 0.35	BF_10_ = 223
TMT B-TMT A	174.1 (67.0)	176.3 (56.8)	185.2 (52.2)	BF_10_ = 0.23	-	-	-
Digit Symbol Test	21.0 (13.6)	22.7 (11.8)	19.4 (12.3)	BF_10_ = 0.08	-	-	-

Mean (standard deviation) and group comparison by Bayesian ANCOVAs including covariates for age, sex, years of education, and time between last clinical visit and autopsy. If the overall model indicated at least moderate evidence for a difference between groups (BF10 > 3), post-hoc pairwise comparisons were performed

AD, Alzheimer’s disease; BF, basal forebrain; BF_10_, Bayes factor in favor of H1 over H0; CDR SOB, Clinical Dementia Rating sum of boxes; LBD, Lewy body disease; MMSE, Mini Mental State Examination; TMT, Trail Making Test

**Table 5:** Effect of tau, amyloid, and Lewy body pathology on cognition

	Braak	Braak +Lewy	Braak +CERAD	Braak +Lewy +CERAD	CERAD	Lewy +CERAD	Lewy
CDR SOB	1.6*10^11^	6.8*10^10^	3.7*10^10^	1.6*10^10^	152.0	24.7	0.11
MMSE	1.9*10^5^	2.4*10^4^	6.1*10^4^	7793	110.4	11.8	0.14
Boston Naming	3.2*10^10^	4.5*10^9^	5.8*10^9^	7.5*10^8^	2316	552.4	1.0
Immediate recall	1.1*10^13^	3.0*10^12^	7.2*10^12^	2.2*10^12^	4.7*10^5^	6.5*10^4^	0.11
Delayed recall	4.1*10^14^	1.2*10^14^	6.4*10^13^	1.9*10^13^	5469	738.3	0.12
Animals	2.8*10^6^	4.2*10^5^	1.2*10^6^	1.9*10^5^	180.7	25.0	0.11
Vegetables	1.3*10^6^	1.1*10^6^	2.4*10^5^	2.1*10^5^	27.6	8.8	0.16
TMT B-TMT A	0.15	0.09	0.04	0.02	0.37	0.18	0.50
Digit Symbol Test	17.2	2.4	2.0	0.28	0.51	0.06	0.11

Bayes factors quantifying evidence in favor of H1 over H0 (BF_10_) from Bayesian ANCOVAs across all groups with the respective cognitive score as dependent variable and the three neuropathological staging systems (Braak stage, CERAD score, presence of Lewy body pathology) as predictors including covariates for age, sex, years of education, and time between clinical visit and autopsy

MMSE, Mini Mental State Examination; SOB, sum of boxes; TMT, Trail Making Test

## Discussion

In a large sample of autopsy-confirmed AD, LBD, and mixed disease, we investigated neuropathological correlates of cholinergic basal forebrain and hippocampus atrophy and their associations with cognition. Degeneration of the posterior basal forebrain was most severe if both AD and LB pathologies were present and models that only included one of the pathologies only showed weak evidence for an association with basal forebrain volume. We know from previous studies in clinically diagnosed AD dementia and DLB patients [1, 2] and previous autopsy findings [18, 34] that the basal forebrain is vulnerable to both pathological processes. Studies of CSF-based biomarkers have shown that in AD, basal forebrain atrophy is mainly related to tau rather than amyloid pathology [35, 36]. Furthermore, basal forebrain atrophy has been found to be related to the presence of alpha-synuclein pathology in a study using a CSF-based seed amplification assay for alpha-synuclein [37]. In the present study, we aimed to go beyond studying the different pathological processes in isolation and were particularly interested in how basal forebrain integrity is affected if both AD and LB pathology are present. Our findings indicate that the combination of the two types of pathology is particularly detrimental to the cholinergic system. This fits with the clinical picture where patients with mixed disease show a more severe manifestation and faster progression [13, 14], potentially in part due to more severe cholinergic loss in these individuals. Conversely, in light of emerging new treatments targeting basal forebrain degeneration [38], people with mixed disease might show the strongest effects in future clinical trials and eventually benefit most from such treatments.

Anterior basal forebrain volume did not differ between the pathological groups and did not show strong associations with the individual neuropathological scores. Similarly, previous studies in clinically diagnosed AD dementia and DLB have reported atrophy primarily in the region of the NBM as well as its white matter connections that provide the main source of cholinergic input to the cortex [2, 39, 40]. Taken together, these findings indicate that cortically projecting cholinergic neurons in the posterior basal forebrain might be more susceptible to AD and LB pathology with relative sparing of the anterior basal forebrain.

Hippocampus atrophy was more specifically related to AD pathology, in particular to Braak stage, and appeared independent of LB co-pathology in those with mixed disease. This finding is in agreement with previous studies and suggests that if hippocampus degeneration is found in clinically diagnosed DLB patients, this might be mainly due to co-occurring AD pathology [10]. 

While the three pathological groups were similar in terms of overall cognitive performance, individual cognitive domains including memory and language were more affected in the groups with AD pathology compared to the pure LBD group, in line with previous evidence [41]. Braak stage was by far the strongest predictor of cognitive performance. The results from the mediation analyses indicate that part of the association between tau burden and overall cognition as well as domain-specific performance on language and memory tests was mediated by hippocampus volume. In contrast, there was no mediation via posterior basal forebrain volume. Furthermore, even though the presence of LB pathology was clearly related to posterior basal forebrain volume, this in turn was not associated with performance on most cognitive tests. The only domain-specific score that showed an association with posterior basal forebrain volume was the Digit Symbol Test which assesses attention/processing speed, consistent with an involvement of the cholinergic system in attention [42]. LB pathology had a significant direct effect on performance on the Trail Making Test, a test of attention/executive function. Attention and processing speed are among the earliest affected cognitive domains in DLB [43] and relate to LB pathological burden [44]. The present results point towards a dissociation between memory/language function and attention/processing speed where the former is related to AD pathology mediated by hippocampus volume and the latter may be additionally related to LB pathology and the cholinergic system. However, basal forebrain volume did not mediate the effect of LB pathology on attention, suggesting that other factors might play a role. The integrity of cortically projecting cholinergic fibres that originate in the NBM has been shown to be more strongly related to attention than basal forebrain volume itself and might be the missing link between LB pathology and cognition [39]. However, overall the AD pathological burden overshadowed the involvement of LB pathology in cognitive performance in this sample.

### Limitations

This study has some limitations. The NACC dataset has been acquired at different sites with only partly standardized procedures. In particular, MRI data were acquired on different scanners at different field strength. To address this issue, we have performed detailed quality control only retaining participants with good quality MRI data and have included site as a covariate in all analyses. While NACC follows standard operating procedures for neuropathological assessments, the autopsy data come from different laboratories and due to the retrospective nature of the dataset we were limited to assessments included in the NACC neuropath dataset. In particular, there was no assessment of regional pathology scores within the basal forebrain. Another limitation is the relatively long time interval between the last MRI and autopsy for some participants. We included this as a covariate, but cannot rule out the possibility that this has affected the strength of the imaging-pathology associations. Despite the use of a large cohort, the number of pure LBD cases remained relatively small (*N* = 22), reflecting the large degree of AD co-pathology in LBD. This smaller sample size of the pure LBD group may have limited our statistical power to find strong evidence for a difference compared to the other groups.

## Conclusion

In a heterogeneous autopsy sample, AD and LB pathology both contribute to cholinergic basal forebrain degeneration whereas hippocampus atrophy is more specifically related to AD pathology. Cognitive deficits are primarily associated with tau pathology which is partly mediated by hippocampus, but not basal forebrain atrophy.

## Electronic supplementary material

Below is the link to the electronic supplementary material.

Supplementary Material 1

## Data Availability

All data used in this study were obtained from the NACC (https://naccdata.org) to which a data request can be submitted.

## Abbreviations

AD: Alzheimer’s disease
ADRC: Alzheimer’s disease research center
BF: Bayes factor
CDR: Clinical dementia rating
CERAD: Consortium to establish a registry for Alzheimer’s Disease
JASP: Jeffrey’s amazing statistics program
LBD: Lewy body disease
MMSE: Mini mental state examination
NACC: National Alzheimer’s coordinating center
NBM: Nucleus basalis of Meynert
UDS: Uniform data set
WMS-R: Wechsler memory scale – revised
