# Supplementary material for: Association of Alzheimer’s and Lewy body disease pathology with basal forebrain volume and cognitive impairment
Source: Alzheimers Res Ther. 2025 Jan 27;17:28. doi: 10.1186/s13195-025-01678-x (PMC11771035; doi:10.1186/s13195-025-01678-x)
Supplement: Supplementary file 1 — Supplementary Material 1 [file 13195_2025_1678_MOESM1_ESM.pdf]

Supplementary Material: Association of Alzheimer's and Lewy body disease pathology with basal forebrain volume and cognitive impairment

Julia Schumacher[1,2], Stefan Teipel[1,3], Alexander Storch[1,2]

[1] Department of Neurology, University Medical Center Rostock, 18147 Rostock, Germany

[2] Deutsches Zentrum für Neurodegenerative Erkrankungen (DZNE) Rostock-Greifswald, 18147 Rostock, Germany

[3] Department of Psychosomatic Medicine, University Medical Center Rostock, 18147 Rostock, Germany

Contact: julia.schumacher@dzne.de

Supplementary Table S1: **Group comparison of regional brain volumes for left and right hemispheres separately**. Mean (standard deviation) and group comparison by Bayesian ANCOVAs including covariates for age at MRI, sex, years of education, time between MRI and autopsy, and site.

	Pure AD	Pure LBD	Mixed AD/LBD	Overall group comparison	Pairwise comparisons		
					Pure AD vs pure LBD	Pure AD vs mixed	Pure LBD vs mixed
Left hemisphere							
Posterior BF volume	0.22 (0.03)	0.23 (0.03)	0.21 (0.03)	BF_{10}=80.3	BF_{10}=1.1	BF_{10}=36.7	BF_{10}=4.1
Anterior BF volume	0.41 (0.04)	0.41 (0.04)	0.41 (0.04)	BF_{10}=0.05	BF_{10}=0.39	BF_{10}=0.15	BF_{10}=0.26
Hippocampus volume	1.38 (0.23)	1.55 (0.25)	1.38 (0.20)	BF_{10}=20.6	BF_{10}=88.4	BF_{10}=0.11	BF_{10}=184.8
Right hemisphere							
Posterior BF volume	0.29 (0.04)	0.29 (0.04)	0.28 (0.03)	BF_{10}=5.0	BF_{10}=0.62	BF_{10}=2.8	BF_{10}=2.4
Anterior BF volume	0.37 (0.05)	0.38 (0.05)	0.37 (0.04)	BF_{10}=0.43	BF_{10}=1.7	BF_{10}=0.18	BF_{10}=1.8
Hippocampus volume	1.47 (0.25)	1.66 (0.25)	1.49 (0.19)	BF_{10}=24.5	BF_{10}=77.3	BF_{10}=0.13	BF_{10}=299.8

AD, Alzheimer's disease; BF, basal forebrain; BF_{10}, Bayes factor in favor of H1 over H0; LBD, Lewy body disease

Volumes are presented as the ratio between regional grey matter volume and total intracranial volume.

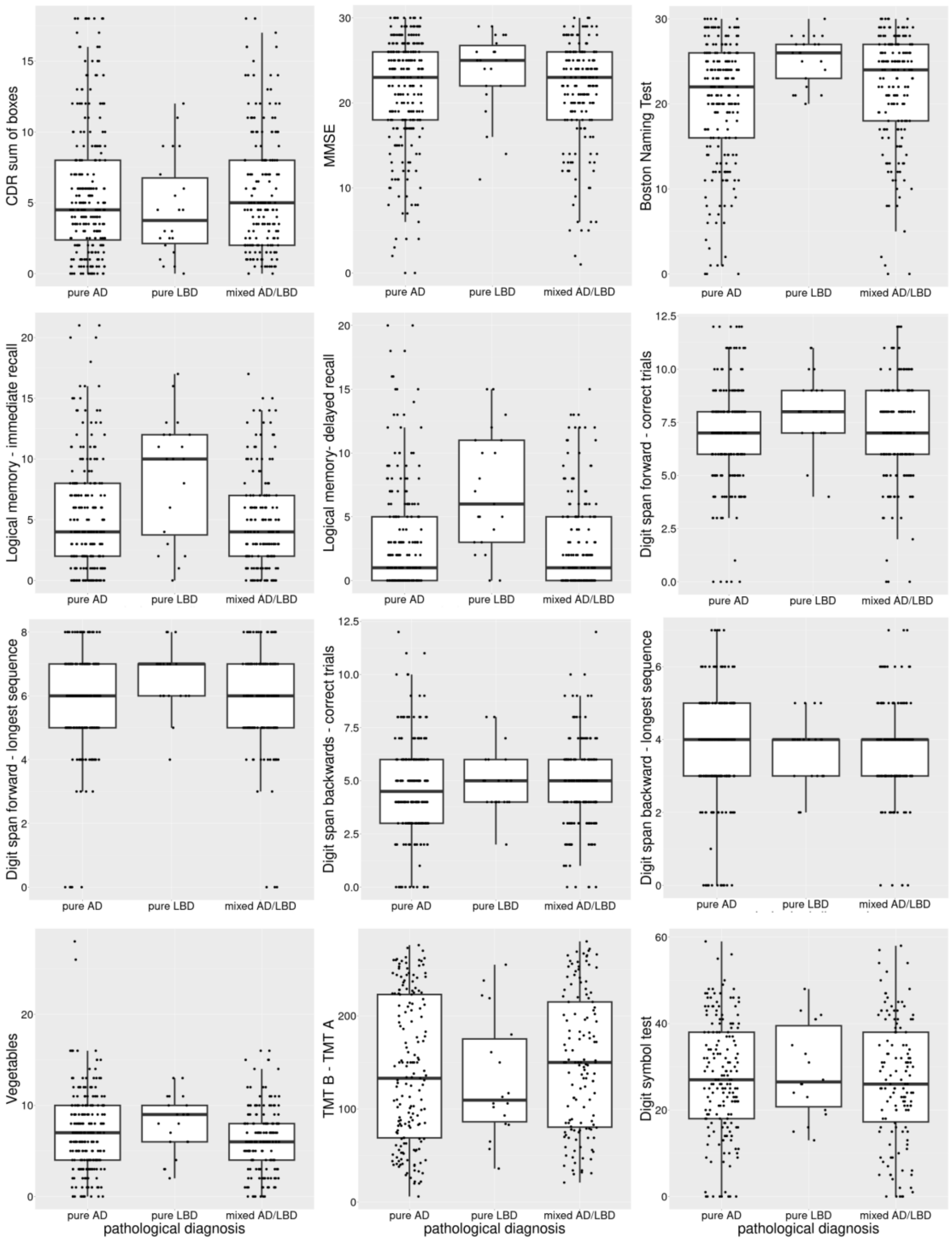

Supplementary Figure S1: Group comparison of cognitive scores. In each box plot the central line corresponds to the sample median, the upper and lower border of the box represent the 25th and 75th percentile, respectively, and the length of the whiskers corresponds to 1.5x the interquartile range. All regional volumes are normalized with respect to total intracranial volume.

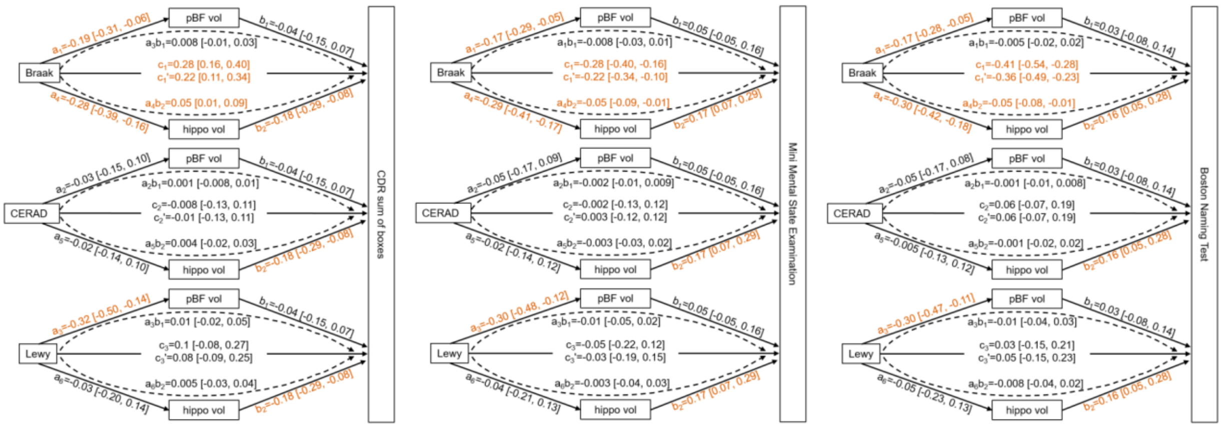

Supplementary Figure S2: One mediation analysis was conducted per cognitive test with three predictors (Braak stage, CERAD score, presence of Lewy body pathology), two mediators (posterior basal forebrain volume, hippocampus volume) and covariates for age, sex, years of education, time interval between MRI and autopsy, and site. 95% credible intervals of the parameter estimates were estimated. Credible intervals that don't overlap with zero are marked in orange.
c=total effect of pathology on cognition, c'=direct effect of pathology on cognition controlling for volume, a=effect of pathology on volume, b=effect of volume on cognition, a*b=indirect effect of pathology on cognition mediated via volume (=c-c').
CDR, clinical dementia rating; hippo vol, normalised hippocampus volume; Lewy, presence of Lewy body pathology; pBF vol, normalised posterior basal forebrain volume.

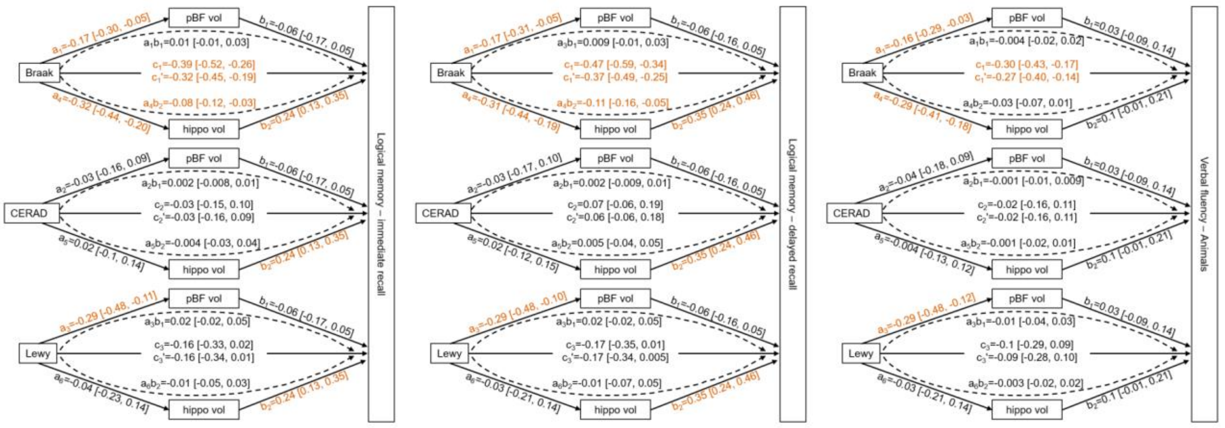

Supplementary Figure S3: One mediation analysis was conducted per cognitive test with three predictors (Braak stage, CERAD score, presence of Lewy body pathology), two mediators (posterior basal forebrain volume, hippocampus volume) and covariates for age, sex, years of education, time interval between MRI and autopsy, and site. 95% credible intervals of the parameter estimates were estimated. Credible intervals that don't overlap with zero are marked in orange.
c=total effect of pathology on cognition, c'=direct effect of pathology on cognition controlling for volume, a=effect of pathology on volume, b=effect of volume on cognition, a*b=indirect effect of pathology on cognition mediated via volume (=c-c').
CDR, clinical dementia rating; hippo vol, normalised hippocampus volume; Lewy, presence of Lewy body pathology; pBF vol, normalised posterior basal forebrain volume.

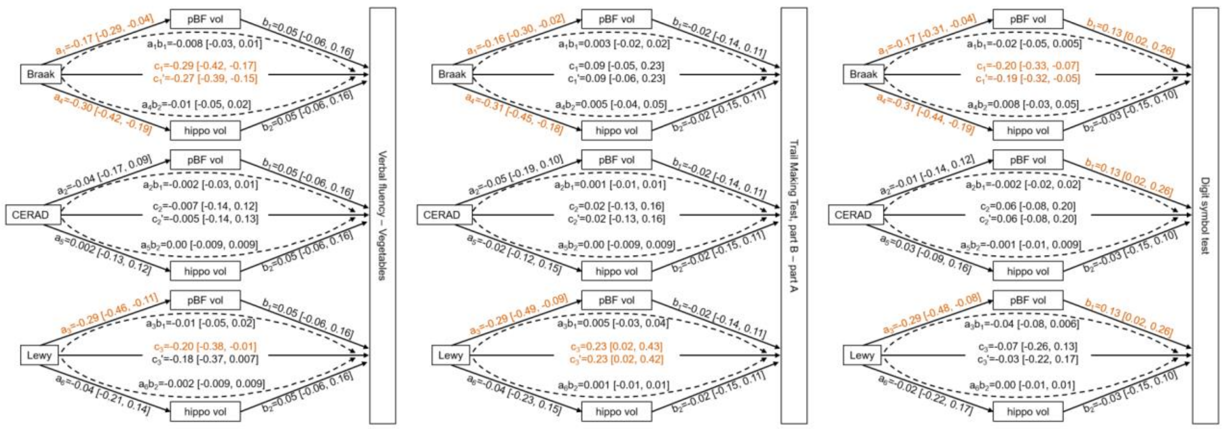

Supplementary Figure S4: One mediation analysis was conducted per cognitive test with three predictors (Braak stage, CERAD score, presence of Lewy body pathology), two mediators (posterior basal forebrain volume, hippocampus volume) and covariates for age, sex, years of education, time interval between MRI and autopsy, and site. 95% credible intervals of the parameter estimates were estimated. Credible intervals that don't overlap with zero are marked in orange.

c=total effect of pathology on cognition, c'=direct effect of pathology on cognition controlling for volume, a=effect of pathology on volume, b=effect of volume on cognition, a*b=indirect effect of pathology on cognition mediated via volume (=c-c').

CDR, clinical dementia rating; hippo vol, normalised hippocampus volume; Lewy, presence of Lewy body pathology; pBF vol, normalised posterior basal forebrain volume.
